# Chromatin dysregulation by mutant p53

**DOI:** 10.18632/oncotarget.7922

**Published:** 2016-03-04

**Authors:** Neil T. Pfister, Carol Prives

**Affiliations:** Department of Biological Sciences, Columbia University, New York, NY, USA

**Keywords:** mutant p53, SWI/SNF, chromatin remodeling, gene expression, VEGFR2

The *TP53* gene, which encodes a DNA sequence-dependent transcriptional regulator, is arguably the most frequently mutated gene in human cancer. Whereas wild-type p53 is restricted to its cognate DNA binding sites, mutant p53 (*via* mutation in the DNA binding domain) is no longer constrained to specific genomic sites. Mutant p53 proteins therefore cannot effectively mediate wild-type p53 tumor suppressive transcriptional programs, thereby enabling permissive tumor growth. Nevertheless, missense mutant forms of p53 can promiscuously alter the transcriptome of the cells they inhabit through association with transcription factors and other chromatin regulators. A global mechanism that could explain mutant p53- dependent gene expression changes would be a useful step in elucidating mutant p53 gain of oncogenic function.

In a recent issue of *Genes & Development*, we reported that mutant p53 stimulates expression of the VEGFR2 receptor tyrosine kinase (RTK), thereby stimulating oncogenic growth and malignant characteristics of breast cancer cells [1]. Augmented transcription of *VEGFR2* was shown to be the result of mutant p53 association with the SWI/SNF chromatin remodeling complex at the *VEGFR2* promoter to enhance SWI/SNF-dependent chromatin remodeling. We further showed that mutant p53 functions with SWI/SNF to mediate >40% of mutant p53-dependent gene expression changes, providing the first evidence that mutant p53 influences promoter conformation and causes global gene expression changes through cooperation with a chromatin remodeling complex [1].

*VEGFR2* was selected as a model gene to study promoter-specific effects of mutant p53 because it is a candidate proto-oncogene and receptor tyrosine kinase with cell signaling functions similar to other known mutant p53-regulated RTKs such as EGFR, IGF1R, MET and PDGFRB (discussed in [1]). Further, wild-type p53 suppresses tumor neoangiogenesis through multiple mechanisms including repression of VEGF and increased degradation of HIF1A [4] and it is often the case that there is an oppositional relationship between genes regulated by wild-type and mutant p53. One implication is tthat cells with wild-type p53 actively antagonize oncogene signaling (a known phenomenon) while cancer cells with mutant p53 actively promote oncogene signaling. Indeed, we found that mutant p53 enhances *VEGFR2* transcription to such an extent that the breast cancer cell lines we tested are dependent on VEGFR2 signaling for optimal growth and cellular migration [8]. We also reported intriguing

clinical trial data suggesting that patients with mutant p53-expressing breast tumors respond better than those with wild-type p53 tumors to anti-VEGF treatment (bevacizumab) although these results would need further statistical validation [1]. We speculate that mutation in p53 coincides with the angiogenic switch, as p53 mutation can simultaneously de-repress VEGF expression and stimulate VEGFR2 expression, which would form a feed-forward system that further stimulates neovascularization and tumor proliferation [3, 1].

While mutant p53 has been known to associate with transcription factors for many years, it has been less clear how mutant p53 association with chromatin leads to changes in gene expression. Using *VEGFR2* as a model mutant p53 promoter, we demonstrated that (1) mutant p53 binds to the *VEGFR2* promoter; (2) mutant p53 restructures the *VEGFR2* promoter into an “open” conformation; (3) mutant p53 binds to the SWI/SNF chromatin remodeling complex at the promoter; (4) mutant p53 depends on SWI/SNF to associate fully with the promoter; and (5) SWI/SNF depletion phenocopies loss of mutant p53 in that SWI/SNF depletion leads to increased nucleosome occupancy, decreased mutant p53 promoter binding, and decreased *VEGFR2* expression [1]. We extended these findings with global gene expression profiling, demonstrating that myriad genes regulated by mutant p53 (both activated and repressed) are co-regulated by SWI/SNF. These experiments establish SWI/SNF as a key effector of mutant p53-mediated transcriptional changes.

**Figure 1:**
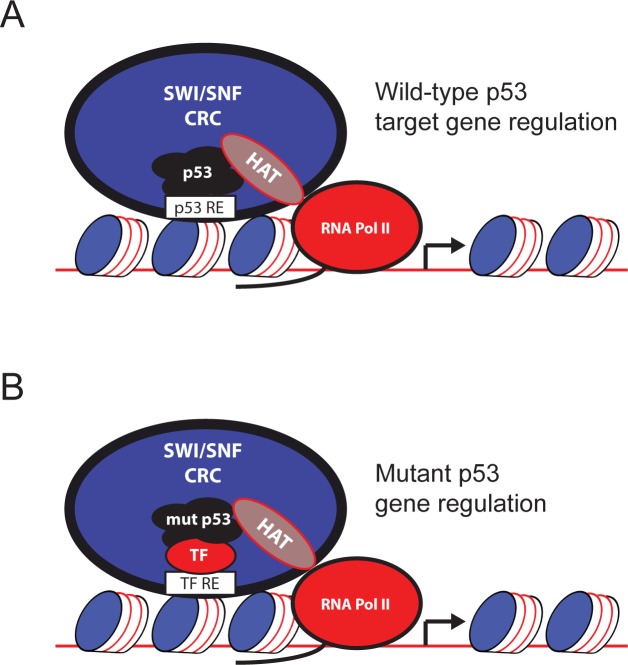
Model for wild-type and mutant p53 gene expression p53 RE, p53 DNA response element. HAT, histone acetyltransferase. TF, transcription factor. TF RE, transcription factor DNA response element. Mut p53, mutant p53. CRC, chromatin remodeling complex. RNA Pol II, RNA polymerase II. **A.** Wild-type p53 target gene expression. Wild-type p53, when activated by cellular stress, binds to its cognate DNA response elements. Subsequently, histone modifiers such as histone acetyltransferases and methyltransferases are recruited to the promoter. SWI/SNF or other chromatin remodeling complexes are also recruited to the promoter and initiate histone displacement, allowing a transcriptionally permissive promoter architecture to form. RNA polymerase II is then activated and initiates transcription of p53 responsive genes. **B.** Mutant p53 gene expression. Mutant p53, which is constitutively active, is not restrained by high affinity DNA binding sites like wild-type p53. Instead, mutant p53 is promiscuous within genomic elements, including with transcription factors and the SWI/SNF chromatin remodeling complex, which can each independently recruit mutant p53 to promoters. Once at the promoter, mutant p53 functions to recruit histone modifiers that can stimulate promoter remodeling by SWI/SNF, causing promoter remodeling that predisposes to dysregulated transcription.

So, how could mutant p53 stimulate SWI/SNF activity? Wild-type p53 is known to functionally interact with multiple histone modifying proteins (eg: p300, CARM1) [4] and chromatin remodeling complexes (including SWI/SNF) [5]. Because mutant p53 cooperates with many of the same proteins as wild-type p53 [6], it is possible that mutant p53 directly stimulates SWI/SNF activity through recruitment of histone acetyltransferases and other histone modifying machinery. As SWI/SNF is known to be more active in the presence of specific histone modifications (reviewed in [7]), and mutant p53 is known to promote histone modifications (see [8] and [9]) mutant p53 recruitment of specific histone modifying machinery likely impacts SWI/SNF activity (Figure 1).

We propose that mutant p53 provides a selective advantage to tumor cells (in addition to the effect of losing wild-type p53 functions) through increasing transcriptional plasticity, which is accomplished in part through cooperation with the SWI/SNF chromatin remodeling complex. The gene expression changes resulting from the presence (usually at high levels) of mutant p53 impact essentially all areas of cancer biology [6]. Recently, mutant p53 was described to impact the transcription of the MLL1 and MLL2 histone methyltransferases and the MOZ histone acetyltransferase, leading to global changes in histone modifications that can facilitate mutant p53 gain of function [10]. It would be interesting to determine the interplay between SWI/SNF-mediated mutant p53 gene expression and the mutant p53 gene expression products that impact transcription, such as MLL1. In any case, we expect the functional interaction of mutant p53 with SWI/SNF to be consistent among tumors, whereas individual gene expression changes mediated by mutant p53 are likely to be stochastic. Targeting cancer-specific chromatin and transcriptional activities is a major therapeutic strategy that is yet to be fully harnessed. Delineation of the mutant p53-SWI/SNF transcriptional mechanism provides a novel strategy to antagonize mutant p53 gain of function.
